# The Burden and Etiology of Community-Onset Pneumonia in the Aging Japanese Population: A Multicenter Prospective Study

**DOI:** 10.1371/journal.pone.0122247

**Published:** 2015-03-30

**Authors:** Konosuke Morimoto, Motoi Suzuki, Tomoko Ishifuji, Makito Yaegashi, Norichika Asoh, Naohisa Hamashige, Masahiko Abe, Masahiro Aoshima, Koya Ariyoshi, Adult Pneumonia Study Group - Japan (APSG-J)

**Affiliations:** 1 Department of Clinical Medicine, Institute of Tropical Medicine, Nagasaki University, Nagasaki, Japan; 2 Department of General Internal Medicine, Kameda Medical Center, Chiba, Japan; 3 Department of Internal Medicine, Juzenkai Hospital, Nagasaki, Japan; 4 Department of Internal Medicine, Chikamori Hospital, Kochi, Japan; 5 Department of General Internal Medicine, Ebetsu City Hospital, Hokkaido, Japan; 6 Department of Pulmonology, Kameda Medical Center, Chiba, Japan; National Institutes of Health, UNITED STATES

## Abstract

**Background:**

The increasing burden of pneumonia in adults is an emerging health issue in the era of global population aging. This study was conducted to elucidate the burden of community-onset pneumonia (COP) and its etiologic fractions in Japan, the world’s most aged society.

**Methods:**

A multicenter prospective surveillance for COP was conducted from September 2011 to January 2013 in Japan. All pneumonia patients aged ≥15 years, including those with community-acquired pneumonia (CAP) and health care-associated pneumonia (HCAP), were enrolled at four community hospitals on four major islands. The COP burden was estimated based on the surveillance data and national statistics.

**Results:**

A total of 1,772 COP episodes out of 932,080 hospital visits were enrolled during the surveillance. The estimated overall incidence rates of adult COP, hospitalization, and in-hospital death were 16.9 (95% confidence interval, 13.6 to 20.9), 5.3 (4.5 to 6.2), and 0.7 (0.6 to 0.8) per 1,000 person-years (PY), respectively. The incidence rates sharply increased with age; the incidence in people aged ≥85 years was 10-fold higher than that in people aged 15-64 years. The estimated annual number of adult COP cases in the entire Japanese population was 1,880,000, and 69.4% were aged ≥65 years. Aspiration-associated pneumonia (630,000) was the leading etiologic category, followed by *Streptococcus pneumoniae*-associated pneumonia (530,000), *Haemophilus influenzae*-associated pneumonia (420,000), and respiratory virus-associated pneumonia (420,000), including influenza-associated pneumonia (30,000).

**Conclusions:**

A substantial portion of the COP burden occurs among elderly members of the Japanese adult population. In addition to the introduction of effective vaccines for *S*. *pneumoniae* and influenza, multidimensional approaches are needed to reduce the pneumonia burden in an aging society.

## Introduction

Globally, pneumonia is a major cause of morbidity and mortality in adults [1, 2]. According to recent estimates, lower respiratory tract infections, including pneumonia, are the fourth most common cause of death, and 1.9 million adults aged ≥15 years die from lower respiratory infections every year [3]. Studies have shown that the risks of pneumonia and pneumonia-related death increase with age and are highest among the elderly [2, 4], indicating that the pneumonia burden is growing in this era of global population aging [5].

Japan is the most aged society in the world; 25% of the Japanese population was aged ≥65 years in 2013 [6, 7]. Although Japanese people have universal access to high-quality medical care as a virtue of universal health insurance coverage [8], an increasing number of elderly people are suffering from pneumonia; the disease is now ranked as the third cause of death in the country. Elucidating the true burden and etiologic fractions is crucial for effective disease control programs; however, the epidemiology of pneumonia remains largely unknown in Japan.

Adult pneumonia has a multi-factorial etiology. *Streptococcus pneumoniae* is the leading cause of adult community-acquired pneumonia (CAP) throughout the world [4, 9, 10], but it has been declining in high-income countries as a result of the wide use of antibiotics and the introduction of pneumococcal vaccines [11]. Meanwhile, non-pneumococcal pneumonia, particularly among elderly people, is gaining attention. Aspiration is considered a major cause of pneumonia in the elderly [12]. The spread of drug-resistant strains is an emerging problem; the risk of drug-resistant pneumonia is particularly high in cases of health care-associated pneumonia (HCAP) and hospital-acquired pneumonia (HAP) [13]. In addition to bacterial pathogens, respiratory viruses (RVs), including influenza and respiratory syncytial virus (RSV), play important roles in the development of pneumonia among the elderly [14]. This variety of etiologies makes controlling pneumonia among the elderly challenging. However, despite improvements in microbiological diagnostic methods, the etiology of pneumonia has not been fully characterized in aged societies.

This prospective multicenter surveillance of adult community-onset pneumonia (COP) covered four major islands in Japan. The study objectives were to establish the age group- and etiology-specific incidences of pneumonia at a population level and to estimate the burden of pneumonia in the entire Japanese adult population.

## Methods

### Ethics

This study was conducted in accordance with the Guideline for Ethical Aspects in Epidemiological Study (Ministry of Health, Labor and Welfare [MHLW], 2008) and was approved by the Institutional Review Boards (IRBs) of the Institute of Tropical Medicine, Nagasaki University, Ebetsu City Hospital, Kameda Medical Center, Chikamori Hospital, and Juzenkai Hospital. Written informed consent was obtained from the majority of the participants or their guardians. The requirement for obtaining written consent from all participants was waived by all IRBs because of the study’s observational nature without any deviation from the current medical practice. Hospital doctors verbally described the study objectives and methods to eligible patients and their guardians during their consultations. We also provided the necessary information to patients and their guardians using a standardized questionnaire sheet and a poster presentation at the outpatient department. Anonymized data were used for the analysis.

### Study setting

According to the national statistics, the total population in Japan was 127 million in 2013 [7]. Of this population, 25.1% were ≥65 years of age, and 3.6% were ≥85 years of age [7]. Although no national recommendation for the 23-valent polysaccharide pneumococcal vaccine (PPV23) existed at the time of study, the cost of PPV23 was partially or fully subsidized by the local government. The estimated coverage rate of PPV23 for adults aged ≥65 years was approximately 25% in 2013 [15].

### Case enrollment

The study was conducted at four community-based hospitals in four prefectures (Hokkaido, Chiba, Kochi, and Nagasaki) in Japan from September 2011 through January 2013. One hospital is located on each of the four main islands (Ebetsu City Hospital in Hokkaido, Kameda Medical Center in Honshu, Chikamori Hospital in Shikoku, and Juzenkai Hospital in Kyusyu). Because of Japan’s universal health insurance system, 70% of the medical costs for people aged <70 years and 80–90% of the medical costs for people aged ≥70 years are covered, regardless of whether the individual is treated in the private or public sector [8]. Therefore, we assume that the characteristics of the pneumonia patients visiting these hospitals do not significantly differ from those visiting neighboring hospitals.

### Enrollment criteria

All the outpatients were screened by hospital physicians, and eligible patients were identified using a standardized case definition. Patients who fulfilled all the following criteria were enrolled in the study: 1) age ≥15 years, 2) with symptoms compatible with pneumonia (e.g., fever, cough, sputum, pleuritic chest pain, and dyspnea), and 3) with new pulmonary infiltrates on chest X-ray (CXR) or computed tomography (CT) scan films that were consistent with pneumonia. To ensure that all eligible cases were enrolled, the study investigators screened the hospital database for International Classification of Diseases, 10th revision (ICD-10) codes and reviewed hospital medical records.

All the enrolled cases were classified into CAP and HCAP groups according to the definitions in the ATS/IDSA guideline [16, 17]. If a patient developed the disease 48 hours after admission, he or she was classified as having HAP and was excluded. Repeated episodes of pneumonia in the same patient within a 2-week period were regarded as a single episode.

### Data collection

Demographic and clinical information were collected from patients and medical charts using a standardized data collection form. Sputum and blood samples were collected from the participants on admission; sputum was induced with the inhalation of hypertonic saline solution if the patients were unable to cough up sputum. CXRs were taken from all patients within 24 hours of admission, and CT scans were ordered by clinicians based on their judgment.

### Microbiological test

Clinical specimens were immediately transported to the laboratory at each hospital. Gram staining was performed on each sputum specimen, and the specimen quality was evaluated by trained laboratory technicians according to Miller and Jones’ classification [18]. All sputum samples were examined using semi-quantitative or quantitative culture methods.

Sputum samples were further tested at the Institute of Tropical Medicine, Nagasaki University, using in-house multiplex polymerase chain reaction (PCR) assays to identify bacterial and viral pathogens. Three typical bacterial pathogens (*S*. *pneumoniae*, *H*. *influenzae*, and *M*. *catarrhalis*), three atypical bacterial pathogens (*Mycoplasma pneumoniae*, *Chlamydophila pneumoniae*, and *Legionella pneumophila*), and thirteen viral pathogens (influenza A virus, influenza B virus, respiratory syncytial virus [RSV], human metapneumovirus [HMPV], human parainfluenza virus [HPIV] types 1–4, human rhinovirus [HRV], human coronavirus [HCoV] 229E/OC43, human adenovirus [HAdV] and human bocavirus) were tested using different sets of multiplex PCR. Details about the primers and PCR methods used have been described previously [19, 20]. We also performed urinary antigen tests for *S*. *pneumoniae* and *L*. *pneumophila* using commercial kits (Binax NOW Streptococcus pneumoniae, Binax NOW Legionella; Alere Inc., Waltham, MA, USA). All pneumococcal isolates were serotyped using the capsular quelling method.

### Etiological category

The etiological category of pneumonia was defined according to the microbiological findings of sputum, blood, and urine samples. The causative pathogens were determined by hospital clinicians and study investigators. Because no culture, PCR or rapid urine test is perfect, we estimated the prevalences of *S*. *pneumoniae* and H. influenzae using two different methods: 1) positivity was defined if either a sputum culture or a urinary antigen test showed a positive result (standard estimation), and 2) positivity was defined if a sputum culture, sputum PCR, or urinary antigen test showed a positive result (maximum estimation).

To estimate the etiology-specific incidence of pneumonia, aspiration-associated pneumonia was defined independent of microbiological profiles. Cases were classified as aspiration-associated pneumonia when the patients had any of the following known risk factors: episodes of aspiration, the presence of dysphagia, consciousness disturbances, neuromuscular diseases, cerebrovascular diseases, tube feeding, and bedridden status [21]. The prevalence of aspiration-associated pneumonia was calculated separately from those of pathogen-specific category.

### The estimation of pneumonia burden

The age-group specific incidence rates of pneumonia, hospitalization and death in the four prefectures were estimated using the surveillance data and the national statistics. We used the pneumonia-outpatient ratio (POR)-based estimation model that was used in our previous study [22]. In this model, the number of age group-specific pneumonia cases was estimated using the age group-specific number of outpatients and the age group-specific POR. PORs were calculated for hospitals and clinics separately. The model used to estimate the number of pneumonia outpatient visits was as follows:

$$I_{ij}\;=\;\frac{\sum_{k}C_{ijk}\times \alpha_{ijk}}{P_{ij}}$$

*I*_*ij*_: the annual incidence of pneumonia in age group i and prefecture j

*C*_*ijk*_: the annual number of outpatients reported to the patient survey in age group i, prefecture j, and facility type k (k = 1, hospitals; k = 2, clinics)

*α*_*ijk*_: the ratio of confirmed pneumonia patients to the number of total outpatients in age group i, prefecture j, and facility type k

*P*_*ij*_: the population in age group i and prefecture j

*C*_*ijk*_ was obtained from the patient survey conducted by the MHLW in 2011 [23]. The survey was conducted at hospitals and clinics on one designated date set for each hospital on one of three days in October. *P*_*ij*_ was obtained from the national demographic survey in 2012 [7].

The proportion of pneumonia cases among outpatients in hospitals (*α*_*ij*1_) was calculated from the surveillance data. To estimate the proportion of pneumonia cases among outpatients in clinics (*α*_*ij*2_), we obtained the clinic databases from two clinics in Nagasaki City. Pneumonia diagnosis was confirmed using the case definition identical to that used for the hospital-based surveillance. *α* was estimated for each age group using curve fitting. Age-standardized rates were calculated using the WHO world standard population [24]. The number of pneumonia cases in the entire Japanese population was estimated assuming that the incidence rates (*I*_*ij*_) were constant across all prefectures.

## Results

### Case characteristics

During the study period, a total of 932,080 patients visited the study hospitals; 1,935 of these patients were enrolled in the study. After excluding 163 cases that did not meet the criteria, 1,772 patients were eligible for our analysis (see supplementary material, S1 Fig). CT scans were administered to 1,332 patients (75.2%), including 110 (8.3%) who did not demonstrate any infiltrates on their CXRs. The demographic and clinical characteristics of the enrolled cases are shown in Table 1. Fifty-nine percent of all patients were male, and the median age was 77 years (range: 15 to 103). Seventy-five percent of the patients were elderly people aged ≥65 years, and 57% were aged ≥75 years. The case fatality rate was 8%. Seventy-two percent of our cases were hospitalized, and these cases were more likely to be male, older, smokers, and classified as having HCAP; more likely to have underlying conditions, aspiration-associated conditions, and severe conditions; more likely to visit the hospital early; and more likely to have a fatal outcome compared with outpatients.

**Table 1:** Demographic and clinical characteristics of patients with community-onset pneumonia in Japan.

	Total	Outpatients	Inpatients	P-value*
	n = 1,772	n = 505	n = 1,267	
Male sex	1,040 (59%)	262 (52%)	778 (61%)	P<0.001
Age group				
15–49 years	202 (11%)	124 (25%)	78 (6%)	P<0.001^†^
50–64 years	242 (14%)	121 (24%)	121 (10%)	
65–74 years	316 (18%)	95 (19%)	221 (17%)	
75–84 years	562 (32%)	111 (22%)	451 (36%)	
85 years and older	450 (25%)	54 (11%)	396 (31%)	
HCAP	572 (32%)	47 (9%)	525 (41%)	P<0.001
With underlying diseases^‡^	1,609 (91%)	389 (77%)	1220 (96%)	P<0.001
With aspiration-associated conditions	677 (38.2%)	52 (10%)	625 (49%)	P<0.001
Current/ex-smoker, n = 1,345	748 (56%)	170 (50%)	578 (57%)	P = 0.023
PPV 23-vaccinated, n = 1,028	356 (35%)	94 (32%)	262 (36%)	P = 0.278
Symptoms >7 days^§^, n = 1,735	255 (15%)	113 (23%)	142 (11%)	P<0.001
Pre-hospital antibiotics, n = 1,742	306 (18%)	83 (17%)	223 (18%)	P = 0.565
CURB65 >3, n = 1,721	182 (11%)	5 (1%)	177 (14%)	P<0.001^¶^
Deceased	140 (8%)	2 (0%)	138 (11%)	P<0.001^¶^

* Characteristics were compared between outpatients and inpatients using chi-square tests unless otherwise indicated.

^†^ A score test for trend was performed.

^‡^ Underlying diseases included diabetes mellitus, hypertension, hyperlipidemia, chronic heart disease, cerebrovascular disease, liver disease, renal disease, collagen vascular disease, chronic lung disease, neuromuscular disease, and malignant disease.

^§^ The period from onset to hospital visit.

^¶^ Fisher’s exact test was performed.

### Microbiology

Among 1,594 sputum samples tested using conventional cultures, causative bacterial pathogens were isolated from 759 (48%) samples; 719 (45%) were monoclonal and 40 (3%) were polyclonal (Table 2). *H*. *influenzae* was the most common bacterial pathogen isolated (10%), followed by *S*. *pneumoniae* (9%). *H*. *influenzae* was more frequently isolated from outpatient cases, while *Staphylococcus aureus*, *Klebsiella pneumoniae*, and *Escherichia coli* were more frequently isolated from hospitalized cases. Among the 718 sputum samples tested using multiplex PCR, 359 (50%) were positive for any bacterial pathogens; 20% were positive for *S*. *pneumoniae*, and 18% were positive for *H*. *influenzae*. *H*. *influenzae* and *M*. *pneumoniae* were more frequently detected in samples from outpatient cases than those from hospitalized cases.

**Table 2:** Microbiological profiles of patients with community-onset pneumonia.

	Total	Outpatients	Inpatients	
	No. positive (%)	No. positive (%)	No. positive (%)	P value*
Sputum culture performed	n = 1,594	n = 417	n = 1,177	
* Haemophilus influenzae*	161 (10)	67 (16)	94 (8)	<0.001
* Streptococcus pneumoniae*	142 (9)	44 (11)	98 (8)	0.17
* Staphylococcus aureus*	121 (8)	16 (4)	105 (9)	0.002
* Moraxella catarrhalis*	90 (6)	31 (6)	59 (5)	0.2
* Pseudomonas aeruginosa*	81 (5)	14 (3)	67 (6)	0.062
* Klebsiella pneumoniae*	54 (3)	3 (1)	51 (4)	<0.001^†^
* Escherichia coli*	36 (2)	3 (1)	33 (3)	0.012^†^
Other bacterial pathogens	113 (6)	7 (1)	106 (8)	<0.001
Any bacterial pathogens	759 (48)	176 (42)	583 (50)	0.01
Sputum bacterial PCR performed	n = 718	n = 236	n = 482	
* S*. *pneumoniae*	146 (20)	51 (22)	95 (20)	0.113
* H*. *influenzae*	130 (18)	63 (27)	67 (14)	<0.001
* M*. *catarrhalis*	98 (14)	33 (14)	65 (14)	0.855
* M*. *pneumoniae*	38 (5)	24 (10)	14 (3)	<0.001
* C*. *pneumoniae*	6 (1)	2 (1)	4 (1)	1^†^
* L*. *pneumophila*	0 (0)	0 (0)	0 (0)	-
Any bacterial pathogens	359 (50)	147 (63)	212 (44)	<0.001
Sputum viral PCR performed	n = 1,201	n = 302	n = 899	
HRV	114 (9)	32 (11)	82 (9)	0.449
Influenza A	64 (5)	13 (4)	51 (6)	0.36
RSV	51 (4)	9 (3)	42 (5)	0.207
HMPV	21 (2)	9 (3)	12 (1)	0.059
Other RVs^‡^	45 (4)	14 (5)	31 (3)	0.347
Any RVs	277 (23)	71 (24)	206 (23)	0.832
Sputum viral and bacterial PCR performed	n = 717	n = 236	n = 481	
Any RVs + any bacterial pathogens	105 (15)	46 (19)	59 (12)	0.01
Blood culture performed	n = 1,039	n = 132	n = 907	
* E*. *coli*	11 (1.1)	0 (0)	11 (1)	0.377^†^
* S*. *pneumoniae*	7 (1)	0 (0)	7 (1)	1^†^
* K*. *pneumoniae*	6 (1)	0 (0)	6 (1)	1^†^
* S*. *aureus*	4 (0)	0 (0)	4 (0)	1^†^
* H*. *influenzae*	2 (0)	0 (0)	2 (0)	1^†^
Urinary antigen test for *S*. *pneumoniae* performed	n = 992	n = 247	n = 745	
* S*. *pneumoniae*	132 (13)	87 (12)	45 (16)	0.121

HRV = human rhinovirus; RSV *=* respiratory syncytial virus; HMPV = human metapneumovirus

* Positive rates were compared between outpatients and inpatients using chi-square tests unless otherwise indicated.

^†^ Fisher's exact test was performed.

^‡^Other RVs included HPIV2 (n = 10), HPIV3 (n = 10), HPIV1 (n = 9), influenza B (n = 6), HCoV (human coronavirus) (n = 4), and HAdV (human adenovirus) (n = 4).

*S*. *pneumoniae* was isolated by blood culture from 2.9% (n = 7/1,039) of cases, and *S*. *pneumoniae* urinary antigen was detected in 13% (n = 132/992). Taken together, 26.2% of samples were positive for *S*. *pneumoniae* either by culture, PCR, or urinary antigen tests. Among 142 *S*. *pneumoniae* isolates, 100 were serotyped; serotype 3 was the most dominant (n = 22, 22%), followed by serotypes 11A (n = 10, 10%) and 19F (n = 8, 8%). PPV23 covered 67% of all serotypes, and 54% were covered by 13-valent pneumococcal conjugate vaccine (PCV13) (see supplementary material, S2 Fig).

Multiplex PCR was used to test 1,201 sputum samples for RVs, and 23% of the samples were positive for at least one RV (Table 2). HRV was the leading virus identified, followed by InfA and RSV. The positivity rates for RVs were similar between the outpatient cases and the hospitalized cases. Among 717 sputum samples tested for viral and bacterial PCRs, 105 (15%) were both positive.

### Aspiration-associated pneumonia

Among all 1,722 patients, 677 (38.2%) were with aspiration-associated conditions (ie, aspiration-associated pneumonia): 373 (21.1%) had episodes of aspiration, 149 (8.4%) dysphagia, 81 (4.6%) consciousness disturbances, 143 (8.1%) neuromuscular diseases, 373 (21.1%) cerebrovascular diseases, 21 (1.2%) tube feeding, and 148 (8.4%) bedridden status. Among 629 sputum samples available from aspiration-associated pneumonia, *Staphylococcus aureus* was the most common bacterial pathogen isolated by culture (10%), followed by *S*. *pneumoniae* (7%) and *Klebsiella pneumoniae* (7%) (see supplementary material, S1 Table). *S*. *pneumoniae* and *H*. *influenzae* were less frequently detected in samples from aspiration-associated pneumonia than those from non-aspiration-associated pneumonia by culture and PCR.

### The burden of COP

**Fig 1:**
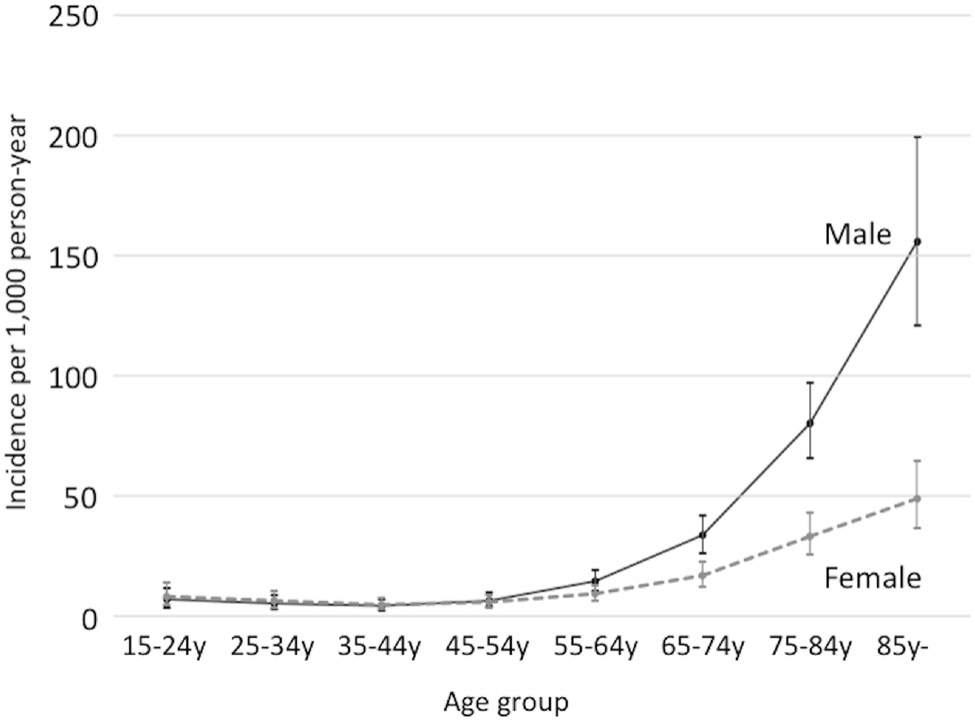
Annual incidences of community-onset pneumonia per 1,000 people by age group and gender. The incidence among the male population is shown as a solid line, and the incidence among the female population is shown as a dashed line. The 95% confidence intervals for each point are shown as vertical lines.

The overall annual incidence rate of adult pneumonia in the four prefectures was 16.9 per 1,000 PY (95% CI, 13.6 to 20.9 per 1,000 PY) in Japan, and the ASR was 10.2 (7.7 to 13.3; Table 3). The incidence rate was highest in Kochi (22.8 per 1,000 PY) and lowest in Chiba (13.7 per 1,000 PY), while the ASR did not differ significantly by prefecture, ranging from 9 to 11.5 per 1,000 PY (see supplementary material, S2 Table). The rate was higher in males than in females (15.6 *vs* 9.3 per 1,000 PY in all age groups; 120.6 *vs* 36.4 per 1,000 PY in people aged ≥85 years). It was lowest in those aged 35–44 years (4.5 per 1,000 PY), increased sharply with age, and became highest in the population aged ≥85 years (79.3 per 1,000 PY; 95%CI, 65.7 to 95.5; Fig 1). This age-associated increase in the rate was more apparent in males than in females (the point estimates of the rate ratios comparing males and females in people aged 55–64 years, 65–74 years, 75–84 years, and 85 years were 1.6, 2, 2.4, and 3.2, respectively). This increasing trend was also observed for pneumonia-related hospitalizations and in-hospital death.

**Table 3:** Estimated annual incidence rates (per 1,000 people) of community-onset pneumonia (COP) in Japanese adults by clinical category, 2012.

Age group	15–24 years	25–34 years	35–44 years	45–54 years	55–64 years	65–74 years	75–84 years	≥85 years	Overall (≥15 years), crude incidence	Overall (≥15 years), age standardized incidence*
	Incidence rate per 1,000 people (95% CI)									
Total COP	7.6 (5.1 to 11)	5.8 (3.8 to 8.3)	4.5 (3 to 6.5)	6 (4.3 to 8.3)	11.8 (9.3 to 14.6)	24.6 (20.4 to 29.4)	52.9 (45.2 to 61.7)	79.3 (65.7 to 95.5)	16.9 (13.6 to 20.9)	10.2 (7.7 to 13.3)
Hospitalization	3.2 (2.2 to 4.7)	1.7 (1.1 to 2.5)	1.4 (1 to 2)	2.5 (1.8 to 3.4)	6.5 (5.1 to 8)	16.9 (14 to 20.3)	43.4 (37.1 to 50.6)	75.4 (62.4 to 90.7)	11.8 (9.7 to 14.3)	5.7 (4.5 to 7.2)
Outpatient	4.4 (2.9 to 6.3)	4.1 (2.7 to 5.9)	3.1 (2.1 to 4.4)	3.5 (2.5 to 4.8)	5.3 (4.2 to 6.6)	7.7 (6.4 to 9.2)	9.5 (8.1 to 11.1)	3.9 (3.3 to 4.7)	5.1 (3.9 to 6.6)	4.4 (3.2 to 6)
In-hospital death	0 (0 to 0)	0 (0 to 0)	0 (0 to 0)	0.1 (0.1 to 0.1)	0.3 (0.3 to 0.4)	0.8 (0.7 to 1)	2.2 (1.9 to 2.4)	6.8 (6 to 7.7)	0.7 (0.6 to 0.8)	0.2 (0.2 to 0.3)
CAP	7.2 (4.8 to 10.4)	5.4 (3.6 to 7.9)	4.1 (2.8 to 6)	5.3 (3.7 to 7.2)	9.5 (7.6 to 11.9)	17.9 (14.8 to 21.4)	33.1 (28.3 to 38.6)	40 (33.1 to 48.1)	11.9 (9.4 to 14.9)	8.1 (6 to 10.7)
HCAP	0.4 (0.3 to 0.5)	0.3 (0.2 to 0.5)	0.4 (0.2 to 0.5)	0.8 (0.5 to 1)	2.2 (1.8 to 2.8)	6.7 (5.6 to 8)	19.8 (17 to 23.1)	39.4 (32.6 to 47.4)	5 (4.2 to 6.1)	2 (1.7 to 2.5)
Aspiration-associated	1.2 (0.8 to 1.7)	0.6 (0.4 to 0.9)	0.5 (0.3 to 0.7)	0.8 (0.6 to 1.1)	2.4 (1.9 to 3)	7.5 (6.2 to 8.9)	22.2 (18.9 to 25.8)	43.4 (35.9 to 52.2)	5.7 (4.7 to 6.9)	2.5 (2 to 3.1)
S pneumoniae-associated, maximum estimate^†^	1.1 (0.7 to 1.6)	0.8 (0.6 to 1.2)	0.9 (0.6 to 1.3)	1.6 (1.1 to 2.2)	3.8 (3 to 4.8)	8.7 (7.2 to 10.4)	16.9 (14.5 to 19.7)	15.7 (13 to 18.9)	4.7 (3.9 to 5.8)	2.6 (2 to 3.3)
S pneumoniae-associated, standard estimate^‡^	1 (0.6 to 1.4)	0.9 (0.6 to 1.3)	0.8 (0.5 to 1.2)	1.2 (0.8 to 1.6)	2.4 (1.9 to 3)	5.1 (4.2 to 6.1)	10.9 (9.3 to 12.7)	15.9 (13.2 to 19.1)	3.3 (2.7 to 4.1)	1.9 (1.4 to 2.4)
H influenzae-associated, maximum estimate^§^	2.5 (1.6 to 3.5)	1.6 (1.1 to 2.4)	1.3 (0.8 to 1.8)	1.7 (1.2 to 2.4)	3.4 (2.7 to 4.3)	6.7 (5.6 to 8)	11.1 (9.5 to 13)	6.7 (5.5 to 8)	3.8 (3 to 4.8)	2.7 (2 to 3.5)
H influenzae-associated, standard estimate^¶^	0.8 (0.5 to 1.1)	0.8 (0.5 to 1.1)	0.7 (0.4 to 1)	0.9 (0.6 to 1.2)	1.6 (1.2 to 1.9)	2.9 (2.4 to 3.5)	5.9 (5.1 to 6.9)	9.4 (7.8 to 11.3)	2 (1.6 to 2.5)	1.3 (0.9 to 1.6)
RV-associated	1.9 (1.3 to 2.8)	1 (0.7 to 1.5)	0.7 (0.5 to 1)	0.9 (0.7 to 1.3)	2.1 (1.7 to 2.6)	5.3 (4.4 to 6.3)	13.2 (11.2 to 15.3)	21.3 (17.7 to 25.7)	3.8 (3.1 to 4.7)	2.2 (1.6 to 2.8)
Atypical bacteria-associated	2.5 (1.7 to 3.7)	2.2 (1.4 to 3.1)	1.8 (1.2 to 2.5)	0.9 (0.7 to 1.3)	0.3 (0.3 to 0.4)	0.7 (0.6 to 0.9)	1.1 (1 to 1.3)	2.4 (2 to 2.9)	1.4 (1 to 1.9)	1.6 (1.1 to 2.3)
PDR pathogen-associated^#^	0 (0 to 0.1)	0 (0 to 0.1)	0.1 (0.1 to 0.1)	0.2 (0.1 to 0.3)	0.6 (0.5 to 0.8)	1.8 (1.5 to 2.2)	5.1 (4.3 to 5.9)	9 (7.5 to 10.8)	1.3 (1 to 1.5)	0.5 (0.4 to 0.6)
Influenza-associated	0.3 (0.2 to 0.5)	0.4 (0.2 to 0.5)	0.3 (0.2 to 0.4)	0.3 (0.2 to 0.4)	0.4 (0.4 to 0.6)	0.8 (0.6 to 0.9)	2.3 (1.9 to 2.6)	6.3 (5.3 to 7.6)	0.8 (0.7 to 1)	0.5 (0.4 to 0.6)

CAP = community-acquired pneumonia; CI = confidence interval; HCAP = health care-associated pneumonia; PDR = potentially drug-resistant; RV = respiratory virus.

*The WHO standardized population was used.

^†^Positivity was defined when a sputum culture, sputum PCR, or urinary antigen test showed a positive result.

^‡^Positivity was defined when a sputum culture or urinary antigen test showed a positive result.

^§^Positivity was defined when a sputum culture or sputum PCR showed a positive result.

^¶^Positivity was defined when a sputum culture showed a positive result.

^#^PDR bacterial pathogens, including methicillin-resistant *Staphylococcus aureus*, extended spectrum beta lactamase-producing gram-negative bacilli, *Pseudomonas aeruginosa*, and *Stenotrophomonas maltophilia*

For the etiology-specific incidence estimates, the etiology-associated incidence rate was calculated according to age group (Table 3). Although aspiration-associated pneumonia had the highest incidence, there were substantial numbers of infection-associated pneumonia cases among older people; *S*. *pneumoniae*-, *H*. *influenzae*-, and RV-associated pneumonia followed. The exception was atypical pneumonia; the incidence of atypical bacteria-associated pneumonia was highest among younger people (aged 15–54 years). In our study population, only 7 out of 1,039 cases had positive blood cultures, indicating that the incidence of bacteremic pneumococcal pneumonia was 12 per 100,000 PY (95% CI, 9.8 to 14.5 per 100,000 PY).

**Fig 2:**
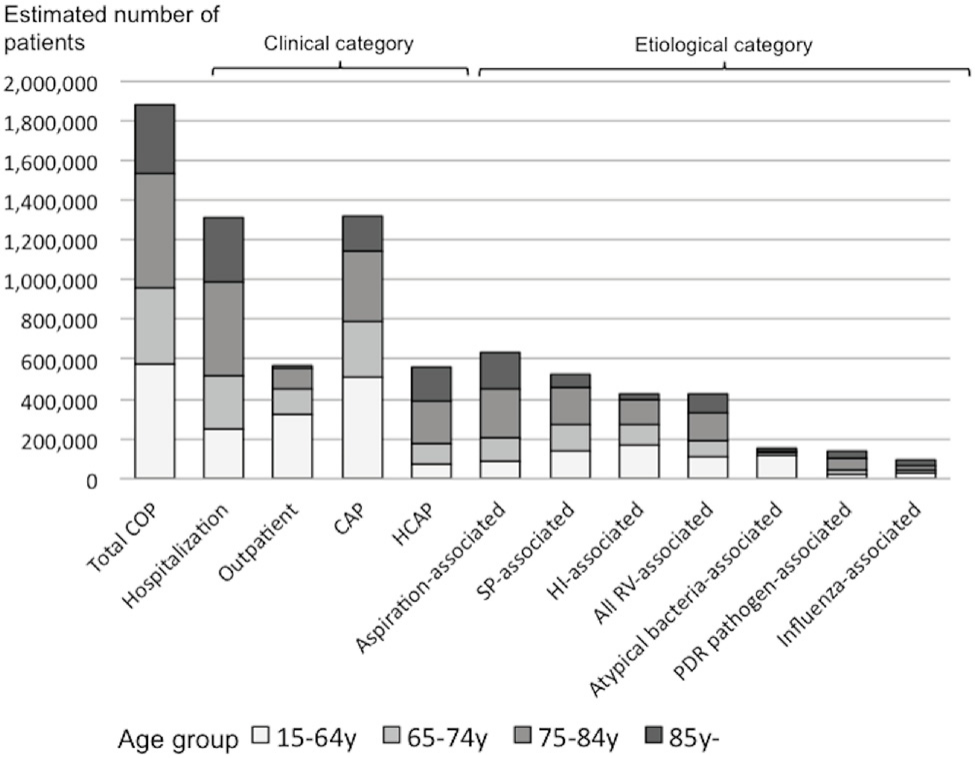
Estimated annual burden of community-onset pneumonia in Japanese adults by clinical and etiological category, 2012. CAP = community-acquired pneumonia; CI = confidence interval; HCAP = health care-associated pneumonia; HI = *H*. *influenzae*; PDR = potentially drug-resistant; RV = respiratory virus; SP = *S*. *pneumoniae*. Maximum estimates are shown for SP- and HI-associated pneumonia.

Assuming that these proportions of pneumonia etiologies were constant across all prefectures, the estimated annual number of COP in the entire Japanese adult population was 1,880,000; of these, 1,300,000 cases (70%) occurred in people aged ≥65 years (Fig 2). Seventy percent were hospitalized cases, and 70% of all pneumonia cases were CAP. Among COP cases, 74,000 died in hospitals. 630,000 cases were aspiration-associated, and 90% of patients with this condition were aged ≥65 years. 530,000 cases were *S*. *pneumoniae*-associated, 420,000 were *H*. *influenzae*-associated, and 420,000 were RV-associated pneumonia.

## Discussion

To our knowledge, this study is the first to estimate the burden of pneumonia in the entire Japanese adult population. In 2012, the overall incidence of community-onset pneumonia, including CAP and HCAP, among people aged ≥15 years was 16.9 per 1,000 PY; the incidence sharply increased with age and reached up to 79.3 per 1,000 PY among people aged >85 years. The estimated annual number of adult COP patients in Japan was 1,880,000, 70% of which were elderly people aged ≥65 years. Aspiration was the leading etiologic category of pneumonia, though a substantial number of cases were still associated with infections, such as *S*. *pneumoniae*. Our findings clearly indicate that pneumonia is an age-related disease that causes an enormous burden in this aging population.

### The burden of COP in the elderly

The incidence of pneumonia in Japanese elderly people was higher than the incidences observed in large-scale population-based studies in both the United States and European countries. In Japan, the incidence of COP among people aged ≥65 years was 42.3 per 1,000 PY; in contrast, COP incidences were 28.4 per 1,000 PY in the United States [25], 14 per 1,000 PY in Spain [26], and 8 per 1,000 PY in the United Kingdom [27]. The high pneumonia incidence in Japan may be partially explained by its high proportion of extremely elderly people aged ≥85 years (14% of the elderly population in 2012 [7]). However, the trend did not fundamentally change after the incidence was standardized using the WHO world standard population: the ASRs of COP among the elderly in Japan, the United States, Spain, and the United Kingdom were 37.1, 25.3, 13.5, and 7.2 per 1,000 PY, respectively. The in-hospital mortality rate for COP in Japan (11.5%) was lower than that reported for other countries (12.4% in the United States [25], 15% in Spain [26], and >25% in the United Kingdom [28]). These findings suggest that patients with mild pneumonia cases that may be overlooked in other countries are visiting clinics and being diagnosed in Japan. In fact, 75% of our pneumonia cases were examined with CT scans to diagnose pneumonia. The good access to healthcare facilities resulting from universal health coverage and the wide use of sensitive diagnostic tools may explain this high incidence. However, it does not indicate that the hospitalized cases in Japan are milder than those in other countries; the proportion of severe cases (CURB65 score ≥3) in the current study was 30%, compared with 38% in the United Kingdom [29] and 10% in Spain [30].

The pneumonia incidence and in-hospital morality were higher among males than among females, especially among the older age group, confirming previous works [4, 25, 26]. Considering the higher incidence of childhood pneumonia among males, they may be genetically vulnerable to pneumonia; however, there is no evidence to support this hypothesis.

### Etiologic fractions and pneumonia control

According to our estimates, aspiration was the leading cause of pneumonia, and the burden of pneumonia associated with aspiration was higher than that associated with any single pathogen, including *S*. *pneumoniae*. The burden was particularly high among the elderly population; 85.8% of aspiration-associated pneumonia cases occurred in patients aged ≥65 years. The in-hospital mortality for aspiration-associated pneumonia (10.9%) was higher than that for other pneumonia categories (6%).

Aspiration-associated pneumonia has been overlooked in current pneumonia control programs. Although previous studies have shown that this condition is common among hospitalized pneumonia patients [2, 31], its burden has never been evaluated at the population level in the past. Aspiration-associated pneumonia is a multi-factorial condition observed in older people. Impaired swallowing and an abnormal cough reflex increase the risk of oropharyngeal aspiration; the aspiration of colonized pathogens and gastric acid causes lower respiratory tract infection and/or lung injury [12]. Compromised immunity, comorbidity and changes in lung function in this age group underlie this condition and are associated with the high mortality. Nursing home residents are at high risk for aspiration, but HCAP and aspiration-associated pneumonia are not identical conditions. In fact, in our study, 25.4% of CAP and 64.3% of HCAP cases were associated with aspiration.

Effective clinical management and preventive measures targeting aspiration-associated pneumonia remain underdeveloped. ATS guidelines recommend using β-lactam/β-lactamase inhibitors for this condition [1, 13], but the management of recurrent and refractory cases is challenging. For prevention, oral hygiene care and dysphagia rehabilitation have been suggested for reducing the risk of aspiration pneumonia, but with limited supporting evidence [32]. The burden of aspiration-associated pneumonia may further increase as the number of elderly people who require long-term care increases. Effective clinical and public health intervention measures are urgently needed.

In the current study, *S*. *pneumoniae* was the leading single etiological pathogen and was associated with 20–28% of pneumonia, confirming previous reports [4]. Recent studies in Japan have shown that the positivity of *S*. *pneumoniae* among CAP cases was 17% [33] to 24% [34]. According to a recent meta-analysis, the proportion of pneumococcal pneumonia among CAP cases was 26–28% [9]. The proportion of pneumococcal pneumonia among all pneumonia cases is declining in high-income countries, reflecting the wide use of antibiotics and pneumococcal vaccines [11]. In our study, the positivity of *S*. *pneumoniae* by sputum culture was only 9%. Considering the low sensitivity of sputum culture, we included urinary antigen test-positive cases for the standard estimation and further included PCR-positive cases for the maximum estimation. The true value must lie between these values (i.e., 20 to 28%). The proportion of bacteremia among pneumococcal pneumonia cases was 6% in our study. A meta-analysis showed that approximately 25% of pneumococcal pneumonia is bacteremic [9]; our figure was lower than this estimate. However, our results showed that the incidence of bacteremic pneumococcal pneumonia among Japanese adults was 12 per 100,000 PY, a figure that was comparable with those reported for other countries, such as the United States [35] and Australia [36]. The findings suggest that pneumococcal pneumonia, either bacteremic or non-bacteremic, remains the leading target for pneumonia control programs in Japan.

PPV23 reduces the risk of invasive pneumococcal diseases (IPDs) among adults; however, its effectiveness against pneumococcal pneumonia is still controversial, particularly for the elderly [37]. The recently approved PCV13 is expected to prevent almost half of the pneumococcal pneumonia cases in the elderly [38, 39]; however, the vaccine covers only 13 serotypes of pneumococcus, and its long-term effects remain unknown. In Japan, before the introduction of PCV7 for children in 2010, 85% of IPD isolates were PPV23 serotypes, and 62% were PCV13 serotypes [40]. In the current study, 67% of the isolates were PPV23 serotypes, and 54% were PCV13 serotypes. The vaccination policy for pneumococcus has been dramatically changing in Japan. PCV7 for children was replaced by PCV13 in late 2013, and PPV23 was also included in the Ministry of Health, Labour and Welfare recommended vaccines for elderly people in late 2014. The proportion of vaccine-covered serotypes is known to decline after widespread use of PCV [41]; thus, these figures will decrease in coming years. The true efficacy of PCV13 for adult pneumonia among the Japanese population must be evaluated along with cost-effectiveness analyses before it is introduced into the national immunization program.

A substantial proportion of pneumonia was associated with RVs (23% of all pneumonia cases). Recent studies suggest that RVs play crucial roles in the development of pneumonia, including severe cases [10, 22, 42–44]; however, their biological mechanisms remain largely unknown. RVs such as influenza, RSV, and human metapneumovirus (HMPV) cause outbreaks among the elderly in nursing homes [45, 46], and these RVs are potential targets for vaccination. Currently, only seasonal influenza vaccines are available for adults, but their effects on pneumonia prevention have not yet been established [47]. Further investigations are needed to clarify the public health impact of RV-associated pneumonia in aging societies.

Our findings have important implications for effective pneumonia control programs in the aging society. The burden of pneumonia is higher in older people, and the pneumonia etiology largely varies by age group: the incidences of aspiration-, *S*. *pneumoniae-*, *H*. *influenzae-*, RV-, and PDR pathogen-associated pneumonia increase with age, while the incidence of atypical bacteria-associated pneumonia decreases. It must be noted that the proportion of pneumonia caused by unknown pathogens is higher among elderly people. This category most likely represents multifactorial conditions. Therefore, in coming decades, the pneumonia burden will likely increase, and its etiology will become more diverse. In this situation, the current etiology-specific approach (i.e., vaccinations for pneumococcus and influenza, guidelines for appropriate antibiotics use) must have only a limited impact. A multidimensional approach integrating vaccination programs, clinical management guidelines, training for health care workers, and education for people must be needed; further studies are warranted.

### Estimation of the pneumonia burden

This study is the first to estimate the national burden of COP in Japan. Although pneumonia is a common disease, its true burden remains unclear, even in high-income countries. A number of studies have reported the incidence of adult pneumonia, but their estimates substantially varied from setting to setting [4, 22, 25–28, 30, 48, 49]. Several factors explain this variation. First, the definition of pneumonia differs among studies. Some studies have reported incidences of CAP that include outpatients and hospitalized patients [25, 30, 49], while other studies have reported hospitalized cases only [28, 48]. It was not clear whether these studies included HCAP cases. Additionally, the diagnosis of pneumonia is not standardized in clinical settings; thus, the burden estimates based on existing database are unreliable. Second, study designs vary. Pneumonia is a common disease, and it is not included in national surveillance. Cohort studies may not represent the entire population of a country, while hospital-based studies do not capture all the cases in the community. Different designs may produce different estimates in an identical population. Third, the health care-seeking pattern affects the incidence estimates. Mild cases must be overlooked in countries in which access to health care is limited. In the current study, we enrolled pneumonia cases prospectively, and all were confirmed by study clinicians using the standardized case definition. Considering the high reliability of national statistics in Japan, our estimates can be reasonably assumed to reflect the true pneumonia burden among Japanese adults.

### Limitations

Our study has limitations. For the incidence estimation, we assumed that the pneumonia-outpatient ratios in the study hospitals were constant across all hospitals in the four prefectures. Additionally, to calculate the national burden, we assumed that the incidence of COP in the four prefectures was constant across all prefectures. Our hospitals were community-based general hospitals that provided primary, secondary, and tertiary care for residents; thus, the patients visiting these hospitals reflected the general population. In fact, the ASRs in the four prefectures were almost identical in our study. Furthermore, according to the national patient survey, the proportion of reported acute respiratory infections among all outpatients in the four prefectures was identical to the national average (see supplementary material). We believe that these assumptions are reasonable. In contrast, we did not consider seasonal differences in the pneumonia etiology. Our etiology-specific burden estimates must be confirmed using multi-year surveillance data. In this study, aspiration-associated pneumonia was defined based on the presence of known risk factors; thus, we could not distinguish between “aspiration pneumonia” caused by aspiration of colonized oropharyngeal pathogen or “aspiration pneumonitis” caused by aspiration of gastric contents [12]. However, there is no reliable marker to identify aspiration [2]. Further studies are needed to better define this pneumonia category.

### Conclusion

A substantial portion of the COP burden in the Japanese adult population occurs in the elderly. Aspiration was the leading etiology of pneumonia, followed by *S*. *pneumoniae*. In addition to the introduction of vaccines for *S*. *pneumoniae* and influenza, multidimensional approaches are urgently needed to reduce the pneumonia burden in this aging society.

## Supporting Information

S1 Database Database of community-onset pneumonia cases. (XLSX)

S1 Fig Flow chart of the study patients. CAP = community-acquired pneumonia; HCAP = health care-associated pneumonia.
(TIF)

S2 Fig Serotype distribution of 100 sputum *Streptococcus pneumoniae* isolates. NT = nontypable.
(TIFF)

S1 Table Microbiological profiles of patients with community-onset pneumonia with and without aspiration-associated conditions. (DOCX)

S2 Table Annual incidence of community-onset pneumonia (per 1,000 people) by prefecture, 2012. (DOCX)

S3 Table The proportion of each category among all community-onset pneumonia (COP) cases by age group. (DOCX)

S4 Table Data dictionary file of database of community-onset pneumonia cases. (DOCX)
