# Supplementary figures and images for: The Burden and Etiology of Community-Onset Pneumonia in the Aging Japanese Population: A Multicenter Prospective Study
Source: PLoS One. 2015 Mar 30;10(3):e0122247. doi: 10.1371/journal.pone.0122247 (PMC4378946; doi:10.1371/journal.pone.0122247)

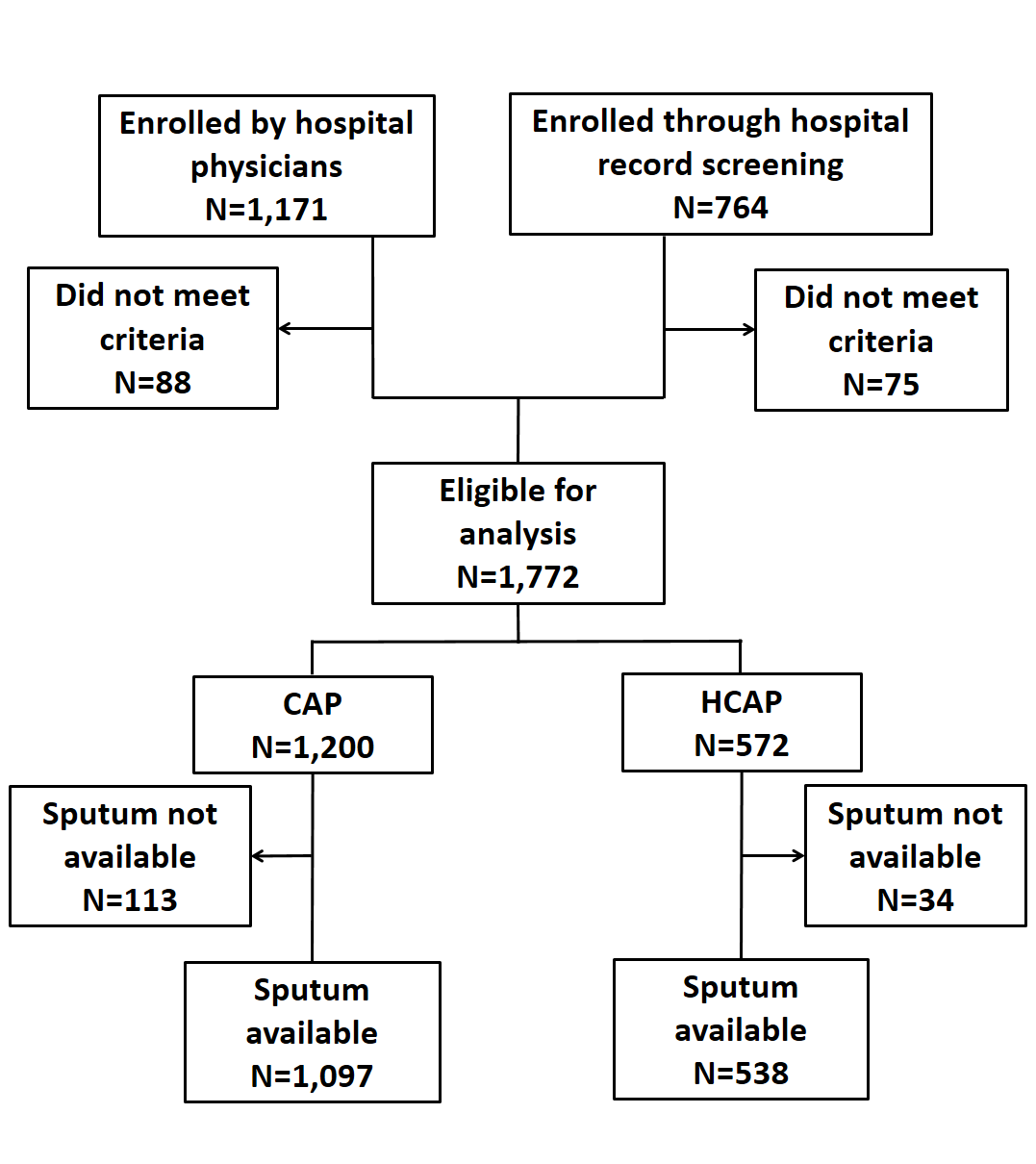

Supplement: S1 Fig — CAP = community-acquired pneumonia; HCAP = health care-associated pneumonia. (TIF) [file pone.0122247.s002.tif]

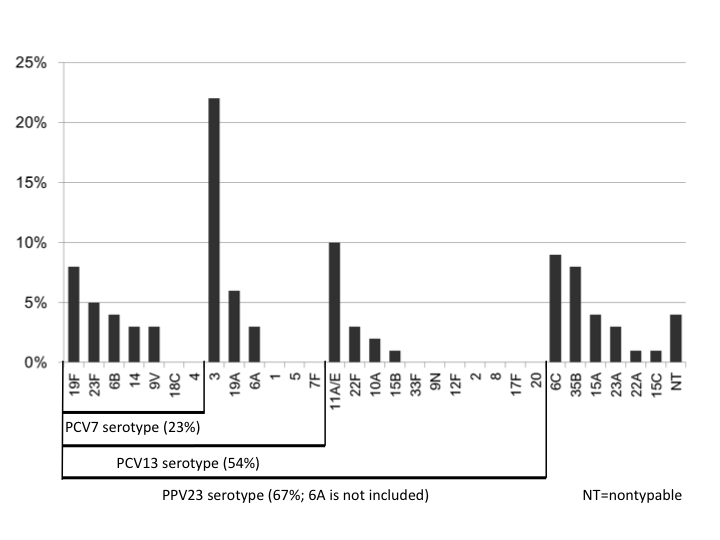

Supplement: S2 Fig — NT = nontypable. (TIFF) [file pone.0122247.s003.tiff]
