# Supplementary material for: The Burden and Etiology of Community-Onset Pneumonia in the Aging Japanese Population: A Multicenter Prospective Study
Source: PLoS One. 2015 Mar 30;10(3):e0122247. doi: 10.1371/journal.pone.0122247 (PMC4378946; doi:10.1371/journal.pone.0122247)
Supplement: S1 Table — (DOCX) [file pone.0122247.s004.docx]

**Table 1.** Microbiological profiles of patients with community-onset pneumonia with and without aspiration-associated conditions

|  | Pneumonia with aspiration-associated conditions, n=677 | Pneumonia without aspiration-associated conditions, n=1,095 |  |
| --- | --- | --- | --- |
|  | No. positive (%) | No. positive (%) | P value* |
| Sputum culture performed | n=629 | n=965 |  |
| *Staphylococcus aureus* | 62 (10) | 59 (6) | 0.006 |
| *Streptococcus pneumoniae* | 45 (7) | 97 (10) | 0.047 |
| *Klebsiella pneumoniae* | 42 (7) | 12 (1) | <0.001 |
| *Pseudomonas aeruginosa* | 37 (6) | 44 (5) | 0.24 |
| *Haemophilus influenzae* | 32 (5) | 129 (13) | <0.001 |
| *Moraxella catarrhalis* | 29 (4) | 61 (6) | 0.231 |
| *Escherichia coli* | 20 (3) | 16 (2) | 0.046 |
|  |  |  |  |
| Sputum bacterial PCR performed | n=267 | n=451 |  |
| *S. pneumoniae* | 42 (16) | 104 (23) | 0.018 |
| *M. catarrhalis* | 41 (15) | 57 (13) | 0.305 |
| *H. influenzae* | 28 (10) | 102 (23) | <0.001 |
| *M. pneumoniae* | 7 (3) | 31 (7) | 0.014 |
| *C. pneumoniae* | 1 (0) | 5 (1) | 0.296† |
| *L. pneumophila* | 0 (0) | 0 (0) | - |
|  |  |  |  |
| Sputum viral PCR performed | n=489 | n=712 |  |
| HRV | 53 (11) | 61 (9) | 0.187 |
| Influenza A | 30 (6) | 34 (5) | 0.303 |
| RSV | 18 (4) | 33 (5) | 0.421 |
| HMPV | 7 (1) | 14 (2) | 0.487 |
| Other RVs‡ | 24 (5) | 21 (3) | 0.079 |
| Any RVs | 122 (25) | 155 (22) | 0.199 |
|  |  |  |  |
| Blood culture performed |  |  |  |
| *E. coli* | 8 (2) | 3 (1) | 0.124† |
| *S. pneumoniae* | 4 (1) | 3 (1) | 0.708† |
| *K. pneumoniae* | 4 (1) | 2 (0) | 0.42† |
| *S. aureus* | 2 (0) | 2 (0) | 1† |
| *H. influenzae* | 0 (0) | 2 (0) | 0.504† |
|  |  |  |  |
| Urinary antigen test for *S. pneumoniae* performed | n=373 | n=619 |  |
| *S. pneumoniae* | 49 (13) | 83 (13) | 0.903 |

HRV=human rhinovirus; RSV*=*respiratory syncytial virus; HMPV=human metapneumovirus

* Positive rates were compared using chi-square tests between two groups unless otherwise indicated.

† Fisher's exact test was performed.
