# Supplementary material for: The Burden and Etiology of Community-Onset Pneumonia in the Aging Japanese Population: A Multicenter Prospective Study
Source: PLoS One. 2015 Mar 30;10(3):e0122247. doi: 10.1371/journal.pone.0122247 (PMC4378946; doi:10.1371/journal.pone.0122247)
Supplement: S2 Table — (DOCX) [file pone.0122247.s005.docx]

**Table 2.** Annual incidence of community-onset pneumonia (per 1,000 people) by prefecture, 2012.

|  | Hokkaido | Chiba | | Kochi | | Nagasaki |
| --- | --- | --- | --- | --- | --- | --- |
|  | Incidence rate per 1,000 people (95% CI) | | | | | |
| Crude incidence, ≥15 years | 17.6 (14.4 to 21.3) | 13.7 (11 to 17) | 22.8 (18.9 to 27.4) | | 21.8 (17.6 to 26.7) | |
| Age standardized incidence*, ≥15 years | 9.8 (7.5 to 12.6) | 9 (6.8 to 11.8) | 11.1 (8.6 to 14.2) | | 11.5 (8.7 to 15) | |

CI=confidence interval.

* The WHO standardized population was used.
