# Supplementary material for: The Burden and Etiology of Community-Onset Pneumonia in the Aging Japanese Population: A Multicenter Prospective Study
Source: PLoS One. 2015 Mar 30;10(3):e0122247. doi: 10.1371/journal.pone.0122247 (PMC4378946; doi:10.1371/journal.pone.0122247)
Supplement: S3 Table — (DOCX) [file pone.0122247.s006.docx]

**Table 3.** The proportion of each category among all community-onset pneumonia (COP) cases by age group.

|  | 15-64 years | 65-74 years | 75-84 years | ≥85 years | Total |
| --- | --- | --- | --- | --- | --- |
| Total COP | 100.0% | 100.0% | 100.0% | 100.0% | 100.0% |
| Hospitalization | 43.4% | 68.8% | 82.1% | 95.0% | 69.9% |
| Outpatient | 56.6% | 31.2% | 17.9% | 5.0% | 30.1% |
| CAP | 88.0% | 72.7% | 62.5% | 50.4% | 70.2% |
| HCAP | 12.0% | 27.3% | 37.5% | 49.6% | 29.8% |
| Aspiration-associated | 15.7% | 30.3% | 41.8% | 54.7% | 33.8% |
| SP-associated | 23.9% | 35.3% | 32.0% | 19.8% | 28.0% |
| HI-associated | 29.4% | 27.3% | 21.0% | 8.4% | 22.6% |
| All RV-associated | 18.5% | 21.5% | 24.8% | 26.9% | 22.6% |
| Atypical bacteria-associated | 20.5% | 3.0% | 2.1% | 3.1% | 8.1% |
| PDR pathogen-associated | 3.0% | 7.5% | 9.6% | 11.3% | 7.4% |
| Influenza-associated | 4.8% | 3.2% | 4.3% | 8.0% | 4.9% |

CAP=community-acquired pneumonia; CI=confidence interval; HCAP=health care-associated pneumonia; HI=*H. influenzae*; PDR=potentially drug-resistant; RV=respiratory virus; SP=*S. pneumoniae*. Maximum estimates are shown for SP- and HI-associated pneumonia.
