# Supplementary material for: The Burden and Etiology of Community-Onset Pneumonia in the Aging Japanese Population: A Multicenter Prospective Study
Source: PLoS One. 2015 Mar 30;10(3):e0122247. doi: 10.1371/journal.pone.0122247 (PMC4378946; doi:10.1371/journal.pone.0122247)
Supplement: S4 Table — (DOCX) [file pone.0122247.s007.docx]

README file:

This file describes the variables in the file of **japan_pneumonia_dataset.xlsx** which was the source of results presented in the paper "The Burden and Etiology of Community-Onset Pneumonia in the Aging Japanese Population: A Multicenter Prospective Study."

If you have any queries, please contact to the corresponding author:

Konosuke Morimoto, MD, PhD

Department of Clinical Medicine,

Institute of Tropical Medicine, Nagasaki University

Sakamoto 1-12-4, Nagasaki, 852-8523, Japan

Email: [komorimo@nagasaki-u.ac.jp](mailto:komorimo@nagasaki-u.ac.jp)

Table 4

| Variable | Type | Label/description |
| --- | --- | --- |
| id | numeric | Record identifier number |
| sex | numeric | 1: male 0: female |
| agegp | numeric | 1: age 15 to 49 years 2: age 50 to 64 years 3: age 65 to 74 years 4: age 75 to 84 years 5: age 85 years and over |
| died | numeric | 1: died 0: survived |
| under | numeric | Presence of underlying diseases 1: Yes 0: No |
| ppv | numeric | PPV23 vaccinated 0: No 1: Yes 2: Unknown |
| symp_durgp | numeric | Duration of symptoms 1: 0-6 days 2: >=7 days 3: Unknown |
| curbgp | numeric | CURB65 score 1: 0-3 2: 4-5 3: Unknown |
| cap | numeric | 1: CAP 2: HCAP |
| smoking | numeric | Smoking status 0: No 1: Yes 2: Unknown |
| preant | numeric | Pre-hospital antibiotics used 0: No 1: Yes 2: Unknown |
| uad_sp | numeric | UAD for *S. pneumoniae* 0: Negative 1: Positive |
| infa | numeric | PCR for influenza A 0: Negative 1: Positive |
| hmpv | numeric | PCR for HMPV 0: Negative 1: Positive |
| rsv | numeric | PCR for RSV 0: Negative 1: Positive |
| rhino | numeric | PCR for HRV 0: Negative 1: Positive |
| anyvirus | numeric | PCR for any RVs 0: Negative 1: Positive |
| othervirus | numeric | PCR for other RVs 0: Negative 1: Positive |
| pcr_sp | numeric | PCR for *S. pneumoniae* 0: Negative 1: Positive |
| pcr_hi | numeric | PCR for *H. influenzae* 0: Negative 1: Positive |
| pcr_mc | numeric | PCR for *M. catarrhalis* 0: Negative 1: Positive |
| pcr_mp | numeric | PCR for *M. pneumoniae* 0: Negative 1: Positive |
| pcr_cp | numeric | PCR for *C. pneumoniae* 0: Negative 1: Positive |
| pcr_lp | numeric | PCR for *L. pneumophila* 0: Negative 1: Positive |
| cult_sp | numeric | Sputum culture for *S. pneumoniae* 0: Negative 1: Positive |
| cult_hi | numeric | Sputum culture for *H. influenzae* 0: Negative 1: Positive |
| cult_mc | numeric | Sputum culture for *M. catarrhalis* 0: Negative 1: Positive |
| cult_sa | numeric | Sputum culture for *Staph. aureus* 0: Negative 1: Positive |
| cult_kp | numeric | Sputum culture for *K. pneumoniae* 0: Negative 1: Positive |
| cult_ec | numeric | Sputum culture for *E. coli* 0: Negative 1: Positive |
| cult_pa | numeric | Sputum culture for *P. aeruginosa* 0: Negative 1: Positive |
| bld_sp | numeric | Blood culture for *S. pneumoniae* 0: Negative 1: Positive |
| bld_hi | numeric | Blood culture for *H. influenzae* 0: Negative 1: Positive |
| bld_ec | numeric | Blood culture for *E. coli* 0: Negative 1: Positive |
| bld_kp | numeric | Blood culture for *K. pneumoniae* 0: Negative 1: Positive |
| bld_sa | numeric | Blood culture for *Staph. aureus* 0: Negative 1: Positive |
